# Medical students’ views: an exploration of medical student attitudes towards disclosure of mental illness

**DOI:** 10.1080/10872981.2020.1792393

**Published:** 2020-07-13

**Authors:** Jawwad Mihran Haider, Fenu Maithriratne Ediripolage, Umar Salim, Syed Kamran

**Affiliations:** Institute of Medical and Biomedical Education, St. George’s, University of London, London, UK

**Keywords:** Medical education, medical students, disclosure, medical school, mental health

We read with great interest the study by Fletcher et al. [1] which delved into non-disclosure of mental illness amongst medical students and their reasoning behind it. We were fascinated by the results, which not only revealed how prevalent intentions not to disclose such information are but also how self-perceived mental health generally deteriorates throughout medical school. Despite this study only being carried out in the University of New Mexico, we strongly believe such attitudes exist universally amongst medical students; a systematic review conducted by the General Medical Council in the UK involving 32 medical schools has shown that many medical students are reluctant to reveal mental health problems, with the evidence suggesting that worries about future careers is a contributing factor [2]. We would like to congratulate the authors on their contributions; as medical students, we recognise the importance of attitudes towards mental health and identifying barriers to seeking help and would like to explore certain areas of the article in greater detail.

The study makes note that, compared to licensed physicians, a far greater proportion of medical students reported that fear of being stigmatized was a reason for non-disclosure of mental illness (65.7% compared to only 5%). Although we agree that increased sensitivity to others’ perceptions early in education is a contributing factor, we wonder whether attitudes of medical students towards individuals with mental illness may play a role too. A study involving medical students in Gujarat, India, highlighted some concerning opinions amongst students regarding mental illness, with 50% of students agreeing that ‘depression occurs in people with a weak personality’ and 57% agreeing that ‘violence mostly results from mental illness.’ [3] As medical students, we have personally observed ill-informed and prejudiced views held by fellow students regarding mental illness, and believe that greater efforts to improve attitudes amongst students towards mental illness will not only reduce stereotyping amongst the future generation of physicians, but also decrease stigmatisation in medical school and encourage students to seek help. We believe that this can be achieved through increased exposure to psychiatric patients in medical school, as previous research corroborates its benefits in relation to altering attitudes [4].

The authors also mention that students may be unfamiliar with the professional consequences of diagnosed mental illness, perceiving them to be worse than they are. Although rumours in medical schools may play a part, we believe that the schools themselves are partly responsible by not thoroughly explaining the implications (or lack thereof) mental illness may have on future careers and dispelling such rumours. A possible solution is the implementation of specialised teaching sessions at the beginning of medical school which clear the air on such issues, granting more students the confidence to seek help by breaking down perceived barriers and preventing deterioration of mental health. Halting such deterioration early on is crucial, as a previous study on medical students has shown that emotional health drops the most between the start and the end of their first year of training; it was also observed that, although emotional health improved after the first year, it never fully returned to baseline illustrating the long-lasting impact such deterioration may have [5].

Furthermore, the study discusses the amendments made by the New Mexico Medical Board to the phrasing of the licensure application. ‘Do you have or have you been diagnosed with an illness or condition which impairs your judgement or affects your ongoing ability to practice medicine in a competent, ethical, and professional manner?’ is the new phrasing utilised in order to reduce stigmatisation compared to the previous wording; however, we believe that this presents a new set of complications.

One major issue we have extrapolated from this question is the fact that it is heavily based on the judgment of the individual who is applying. During our training, we have been educated and instructed on the essential skill of detecting whether our colleagues’ ability to fulfil their duties is declining and the appropriate actions to take in a situation of this sort. This is based on the principle that many physicians and medical students are unable to accurately evaluate their own ability to perform, with colleagues often noticing such changes first. As the authors have correctly identified in the abstract of the article, physicians who are unwell have been shown to ‘deliver lower quality patient care’ and are themselves at increased risk of ‘burnout and suicide’. Therefore, the wording within the licensing application may result in issues stemming from individuals being unable to successfully assess their own situation and we feel that it may be beneficial if more objective phrasing is used in such applications.
